# “Money talks, bullshit walks” interrogating notions of consumption and survival sex among young women engaging in transactional sex in post-apartheid South Africa: a qualitative enquiry

**DOI:** 10.1186/1744-8603-9-28

**Published:** 2013-07-18

**Authors:** Yanga Z Zembe, Loraine Townsend, Anna Thorson, Anna Mia Ekström

**Affiliations:** 1 Health Systems Research Unit, South African Medical Research Council, Cape Town, South Africa; 2 Department of Public Health Sciences/Global Health, Karolinska Institutet, Stockholm, Sweden

**Keywords:** Transactional sex, Young women, Consumption sex, Survival sex, HIV, Wealth inequalities, Post-apartheid, Global technologies, Modernity, South Africa

## Abstract

**Background:**

Transactional sex is believed to be a significant driver of the HIV epidemic among young women in South Africa. This sexual risk behaviour is commonly associated with age mixing, concurrency and unsafe sex. It is often described as a survival- or consumption-driven behaviour. South Africa’s history of political oppression as well as the globalization-related economic policies adopted post-apartheid, are suggested as the underlying contexts within which high risk behaviours occur among Black populations. What remains unclear is how these factors combine to affect the particular ways in which transactional sex is used to negotiate life among young Black women in the country.

In this paper we explore the drivers of transactional sex among young women aged 16–24, who reside in a peri-urban community in South Africa. We also interrogate prevailing constructions of the risk behaviour in the context of modernity, widespread availability of commodities, and wealth inequalities in the country.

**Methods:**

Data were collected through 5 focus group discussions and 6 individual interviews amongst young women, men, and community members of various age groups in a township in the Western Cape, South Africa.

**Findings:**

Young women engaged in transactional sex to meet various needs: some related to survival and others to consumption. In this poverty-stricken community, factors that created a high demand for transactional sex among young women included the pursuit of fashionable images, popular culture, the increased availability of commodities, widespread use of global technologies, poverty and wealth inequalities. Transactional sex encounters were characterized by sexual risk, a casual attitude towards HIV, and male dominance. However, the risk behaviour also allowed women opportunities to adopt new social roles as benefactors in sexual relationships with younger men.

**Conclusion:**

Transactional sex allows poor, young women to access what young people in many parts of the world also prioritize: fashionable clothing and opportunities for inclusion in popular youth culture. In the context of high HIV prevalence in South Africa, strategies are needed that present young women with safer economic gateways to create and consume alternative symbols of modernity and social inclusion.

## Background

Transactional sex, where sex is exchanged for cash and/or material goods and/or alcohol [1-4], is an important risk behaviour for HIV in Africa. Typically, transactional sex occurs between young women and casual male sexual partners who are older and wealthier than they are [2,3,5-8] and who are popularly referred to as sugar daddies. In the patriarchal context that marks many African societies, transactional sexual relationships with sugar daddies compromise young women’s ability to negotiate safe sex practices and increase the risk of male perpetrated intimate partner violence [6,8,9]. Further, multiple, concurrent sexual partnering and low or no condom use often mark these relationships [2,7-11] as these behaviours are thought to maximize the monetary rewards from the practice [1]. In this way, transactional sex exposes women to multiple risk behaviours that they may not have been exposed to in the absence of the practice [3].

Transactional sexual relationships are not a recent phenomenon in Africa, but the ways in which they are defined, named and discussed have evolved and are not uncontested. The literature on transactional sex suggests that the practice of exchanging money or gifts between people in sexual relationships has always been part of normative sexual behaviour in most Black African communities. It is a practice that is driven by the cultural notion that “no self-respecting woman would remain in a friendship without material recompense” [1,12]. Ethnographers travelling the African continent in the 18^th^ century labelled all elements of sexual exchange that they encountered as prostitution [1,2,13] in stark opposition to the informants’ own definitions and reasoning about their behaviour [1]. In doing this, they overlooked two factors: firstly that monetary exchange in sexual relationships can be both “a practical economic arrangement and a symbol of love” [4,12,14], and secondly that transactional sexual partnerships are heterogeneous [3] and generally exist on a continuum, largely defined by time, with relationships lasting a lifetime at the one extreme, or a few minutes of passion at the other [12,15]. Clearly not every form of sexual exchange lends itself neatly to definitions of prostitution.

As with most forms of sexual behaviour, defining transactional sex precisely continues to be a challenge for social scientists. Definitional challenges owe in part to the existence of prostitution on the one hand, and the fact that it is common practice to exchange gifts in sexual relationships in many parts of the world [16,17] on the other. However, contemporary discourse on sexual exchange encounters accepts that they are not prostitution, but transactional sex if: sexual exchange is undertaken within the context of a relationship (no matter how ambiguous or transitory its nature); the negotiation of exchange items is neither explicit nor upfront; and those who engage in the practice determinedly differentiate their behaviour from prostitution [2,13,18,19].

In the context of high HIV prevalence in South Africa, understanding and discussing transactional sex is a strong focus of HIV behavioural research. This is to be expected given the differential vulnerability of heterosexual women to HIV compared to men in the country [20,21]. Further, evidence suggests that transactional sex is a common feature of sexual relationships in the country. For example, in a study among 1645 men aged 18–49, residing in rural, farm and urban areas of South Africa, 66% reported at least one transactional sexual relationship [15]. As with all sexual norms, the practice of transactional sex and its associated risk behaviours have undergone reconstruction, transformed and shaped by the country’s many historical and political processes including colonisation, apartheid, and the forces of globalization that have marked the post-apartheid era [2,14,22,23]. The periods of colonisation and apartheid affected the configuration of sexual relationships in South Africa through the migrant labour system and laws that restricted the movement of Black women to urban areas, which forced men to live apart from their spouses for long periods of time. The separation of men from their rural sexual partners created new demands for formal commercial sex work in urban areas, weakened fidelity in marriage, and advanced the behavioural factors that are linked to transactional sex, namely multiple concurrent sexual partnering [14,24,25], and male biased gender inequalities [14]. Notably, during these periods, premarital sexual exchange relationships, whilst in existence, were not major drivers of sexual behaviour. Instead boyfriends and girlfriends engaged in reciprocal gift giving [26], and men constructed images of masculine success from their ability to save money for *ilobola* (bride-wealth), build a rural homestead, and continue their bloodline through children [14]. Today, owing to high rates of unemployment, and thus a reduced capacity to afford *ilobola*, marriage in Black communities is uncommon and in its absence men have turned to alternative ways of expressing masculine success; namely multiple concurrent sexual partnering and transactional sex [2,14,22].

Several other laws of the apartheid era (e.g. the Group Areas Act No. 41, of 1950; the Bantu Education Act No.47 of 1953 and the Industrial Conciliation Act No. 28 of 1956) marginalized Black Africans from meaningful economic participation, and set in motion the racially and geographically defined wealth inequalities that persist as a major challenge in the country [27]. It is hypothesised that these wealth inequalities are part of the structural factors that give rise to high rates of sexual risk behaviours, such as transactional sex, that are found among Black African men and women in South Africa [14].

Post apartheid, South Africa dismantled apartheid laws and adopted a neo-liberal, Growth, Employment and Redistribution (GEAR) policy. This policy stressed open-ness, trade liberalization, privatization, direct foreign investment, and an emphasis on increased flow of goods (in and out of the country) through less-regulated import and export industries [28-30]. These economic policies incorporated the country into the global economy and culture and brought about a more pronounced presence of globalization, which in this context, is defined as openness [of the world] to trade, information, people, and culture [31]. Research shows that the benefits and the detriments of globalization are experienced differently in the world depending on populations’ socio-economic status and access to resources, among other things.

Further, a few ethnographic studies in South Africa have linked women’s motivations for engaging in transactional sex to globalization. These studies established that proceeds from sexual exchange encounters are used to purchase symbols of global beauty and success that are propagated by the local and global print and visual media [2,5,22]. Combined with other globalization-related consequences of GEAR, such as the massive influx of foreign, cheap fashionable clothing from Asia [32-34], these macro-economic changes have increased the consumption appetites of the poor without creating secure, economic pathways for satiating them. Further, the powerful influence of these macro-economic forces on young women’s sexual behaviour is compounded by their particular stage of psycho-social development, which is characterized by a pre-occupation with outward appearance, role confusion, vulnerability to peer pressure, and propensity for risk taking behaviour [22,23,35].

Little is known about the combined effect of dismantled apartheid laws and the adoption of the globalization-related, post-apartheid macro-economic policies on the consumption behaviours of Black South African people. These transformation processes have essentially moved Black people from socio-economic isolation to [sudden] exposure to commodities and global lifestyles, and may have thus affected their consumption behaviours in unexpected ways. It may be that owing to this unique history of restriction, commodities are accorded far greater importance among Black people than would be the case among other populations.

Importantly, analyses of the post-apartheid, socio-economic context suggest that the inter-racial wealth disparities promoted during the apartheid era have widened, along with significant increases in intra-racial inequality [36]. Since wealth inequalities are important structural influences in the trajectory of risk behaviours [37], it is possible that socio-economic inequalities that are pronounced within a setting that is marked by generalized poverty have a stronger influence on the behaviours of the poor than when wealth is located at a (geographical) distance.

The well-documented influences of South Africa’s political history and the socio-economic arrangements of the present era on sexual behaviour raise important questions about young women’s motivations for engaging in transactional sex. There are studies that suggest that the practice is a survival-driven strategy (survival sex) [11,13,38], the poor woman’s means of “getting by in a world of poverty and disempowerment” [13]. Another school of thought sees transactional sex as a means for young women to fulfil their consumption appetites and pursue images of modernity and success (consumption sex) [2,4,7,39] in the context of globalization. In each of these paradigms, the framing of discussions on transactional sex relies on assumptions that either overplay or underplay the poverty circumstances of women who engage in sexual exchange relationships. Nuanced considerations that explore the interaction between consumption and survival needs are needed to frame young women’s motivations for engaging in transactional sex.

In this paper we explore the drivers of transactional sex among young women aged 16–24, who reside in a peri-urban community in South Africa. We also interrogate prevailing constructions of the risk behaviour in the context of modernity, widespread availability of commodities, and wealth inequalities in the country.

## Methods

### Study setting

The study community is located in the Cape Winelands region of the Western Cape Province, which is located sixty kilometres north of Cape Town, in South Africa. The Western Cape Province is the second wealthiest of the nine provinces that make up South Africa. However, it also has the highest levels of wealth inequalities in the country [40]. Consistent with the distribution of poverty at the national level, the highest incidence of poverty in the Western Cape is found amongst the Black population, where almost half of this population is classified amongst the province’s poorest households and, together with the Coloured (multi-racial) population, account for all abject poverty in the province [41].

The Cape Winelands, within which the study community is located, mirrors the wealth inequalities reported for the province, with poverty pockets scattered in the midst of wealthy neighbourhoods. The study community, a township, is one such poverty pocket. It has a population of 25600 people, many of whom are Black and Xhosa speaking (99%), and unemployed (38%) [42]. It is a by-product of the many segregation laws passed pre- and during apartheid, which made it compulsory for people to reside only in areas declared for the exclusive use of their particular racial group [43].

As with many other townships in post-apartheid South Africa, the community has recently seen the emergence of shopping complexes, large chain store supermarkets, cafés, formal and informal clothing retail stores, creating localized and thus easier access to material goods [44].

### Ethics statement

At the beginning of every focus group discussion and individual interview, information and consent forms were read, discussed and signed with and by each of the participants. Due to the sensitivity of our research questions, parental consent for participants who were younger than 18 years was not sought. Instead the lead author conducted parent information sessions in all the schools in the study community wherein the study background, aims and objectives were presented. No parents objected to the possible inclusion of their underage children in the study. We submitted feedback reports and signed attendance registers to the local ethics committee once the parents’ meetings had taken place.

The Research Ethics Committee of the Faculty of Health Sciences, University of Cape Town, South Africa granted ethics approval to conduct this enquiry.

### Study design

The study made use of an exploratory and emergent qualitative design that employed focus group discussions (FGDs) and individual interviews (IIs) to explore the characteristics and drivers of transactional sex amongst young and sexually active Black women. Data were collected from young Black women aged 16–24 years; Black men aged 20–35 years; and community members of various age groups residing in a peri-urban township in which the study was conducted.

Recruitment and data collection were undertaken in three phases (Table 1). During the first phase (Phase I) FGDs were held with young women who were purposively sampled based on whether they self-reported multiple sexual partnering (FGD 1), or monogamous sexual relationships (FGD 2). Phase II comprised two additional focus group discussions (FGD 3 and 4) with young women who were purposively sampled from alcohol drinking venues known as shebeens, and who self-reported sexual activity in the past 3 months. Phase III comprised one FGD with men (FGD 5) and six IIs with community members who were aged 16 years and older in the late 1980’s to explore whether and in what ways sexual risk behaviours and consumption patterns may have changed over time. All FGDs and IIs were conducted in the local isiXhosa language. Only the first FGD was translated into English; owing to funding limitations, subsequent transcripts were not translated although all the coding for the analysis was done in English.

**Table 1:** Data collection phases

**Dcixata collection phase**	**Data collection method**	**Participants**	**Number of participants**	**Age range**	**Sampling procedure**	**Inclusion criteria**	**Data collector**
I	Focus Group Discussion 1	Young women	10	16-21 years old	Purposive	Resident in the study community, reporting multiple sexual partnering	Lead author (aged 27) and 1 female research assistant aged 23, both Black and Xhosa speaking
I	Focus Group Discussion 2	Young women	10	16-18 years old	Purposive	Resident in the study community, reporting monogamy	Lead author (aged 27) and 1 female research assistant aged 23, both Black and Xhosa speaking
II	Focus Group Discussion 3	Young women	8	16-24 years old	Purposive	Resident in the study community, reporting current sexual activity	Lead author (aged 27) and 1 female research assistant aged 23, both Black and Xhosa speaking
II	Focus Group Discussion 4	Young women	8	16-24 years old	Purposive	Resident in the study community, reporting current sexual activity	Lead author (aged 27) and 1 female research assistant aged 23, both Black and Xhosa speaking
III	Focus Group Discussion 5	Men	6	23-32 years old	Purposive	Aged 20+	2 male research assistants aged 25 and 32 respectively, both Black and Xhosa speaking
III	Individual Interviews 1-6	Men and women	6	36years and older	Purposive	Resident in the study community, aged ≥ 16 years in the late 1980’s	3 female research assistants aged 34, 36 and 40 respectively, all Black and Xhosa speaking

### Formative assessment and data collection

Prior to data collection the lead author and a local, trained, Black, 23 year old female research assistant made several visits to shebeens as part of formative assessment activities. The purpose of the formative assessments was to determine sexual and social norms in the study community and to recruit participants. Based on findings emanating from this process, multiple sexual partnering was set as the inclusion criterion for the first FGD (FGD 1). However, for subsequent FGDs this inclusion criterion was dropped because it was decided that the voices of women who may engage in transactional sex without necessarily self-reporting multiple sexual partnering were also important.

The findings of the formative assessments were used to construct FGD facilitation guides that contained unstructured and open-ended questions about sexual experience, modernity, and motivations for transactional sex. For example, an opening question in the facilitation guide asked: “Tell me about what it means to be a young woman here in the study community”. Another question asked: “What are the signs of modernity among young women in the study community?” These questions were also informed by contemporary literature on transactional sex.

For FGDs 3 and 4 an A4 sized print media image of a young, Black, woman (aged between 20 and 24) dressed in smart yet casual fashionable clothing was passed around and participants were asked to share the most immediate thoughts that came to mind as they studied the picture. The image was used to explore the ideas and importance that young women attach to fashionable images, and how they go about attaining similar images for themselves. Pursuing this particular angle was inspired by the literature on transactional sex and the findings of FGD 1 and 2, which suggested that access to fashionable images is one of the drivers of transactional sex. The idea of using this image was based on the first author’s prior experience with Paulo Freire’s popular education methods (http://www.freire.org) that make use of pictorial material to stimulate critical thinking and discussions about social processes.

### Data analysis

All data were analysed using Graneheim et al’s [45] manifest and latent content analysis methods. The transcripts of FGD 1 and 2 and field notes were analysed during a two-day analysis workshop attended by the lead author, second author and trained research assistants. At the workshop, every member of the research team individually read through each of the transcripts, shared initial thoughts, and began manifest coding of the data. A total of 25 codes was generated, which included “modernity”, “sugar daddies”, “younger sexual partners”, “food”, “alcohol”, “fashionable clothing”, “HIV”, “inequality”, “male power”, “poverty”, “unemployment”, “technology”, “fear of exclusion”, “loneliness” and “being left out”. These were grouped together into categories such as “consumption items”, “subsistence items” etc., that were then further transformed into major themes. Major themes included “motivations for transactional sex”, “local wealth inequalities”, “interaction between age and economic asymmetries and transactional sex”, “consumption sex for survival”, “popular culture”, “forces of globalization”, and “the role of peer pressure”. The codes, categories and themes generated from this analysis were then used in the analysis of the remaining FGDs, which was undertaken by the lead author using the same steps that were followed during the analysis workshop.

### Reflexivity

All necessary steps were taken to ensure adherence to the principles of objective scholarship throughout the collection and analysis of these data. The lead author, a young (27 year old at the time), Black, Xhosa-speaking female PhD student with a rural middle-class background, engaged in continuous reflexivity to ensure an open mind, treating all prior and personally held beliefs, assumptions and knowledge about the study population and the subject matter with awareness, suspicion and distance. However, an open mind is not an empty mind, and since qualitative analysis is interpretative, interpretations are not entirely separate from the researcher’s experiential background [46]. The co-facilitator in FGDs 1–4 was a young (23 year old at the time) Black woman from the study community, with a secondary school qualification and three years experience in field research. Because she was a local woman who resided in the study community, we debriefed regularly and limited her participation to note taking and recruitment. The co-facilitators of the men’s FGD were Black men aged 25 and 32; neither came from the study community, but both were first language speakers of isiXhosa and were fluent in the colloquial “*tsotsi taal*” language commonly used by men in South African townships.

## Findings

### The use of transactional sex to negotiate poverty, local constructs of modernity, and wealth inequalities

Transactional sex was said to be a common feature of young women’s sexual relationships in the study community. Broadly, the young women and men linked the popularity of transactional sex in their community to poverty; local, social constructions of successful sexual relationships; modernity; and their preoccupation with money, fashion, and alcohol. When discussing their transactional sexual exploits, the young women mentioned a wide range of items that were exchanged and/or purchased with proceeds from transactional sex activities. These items included money, clothing, alcohol, food, cellular telephone recharge vouchers, school fees, electricity for their homes, and family meals. Notably, items were mentioned without any attempts to categorize them into degrees of importance:

*He buys you food…and 6% (slang for alcohol)***(FGD 1, young women aged 16–21)**

*Come the 15*^*th*^*of the month (pay day for provincial government employees) he gives me his [bank/retail store] card to go and buy myself a new outfit or a pair of sneakers from Sportscene (an expensive sportswear store)***(FGD 4, young women aged 16–24)**

*You buy paraffin, you take some money home and buy electricity***(FGD 3, young women aged 16–24)**

*You warm up your home, make it warm, even providing [your family with] mngqusho (an African staple food, made with dried corn kernels and beans)*(**FGD 4, young women aged 16–24)**

However, although poverty and wealth inequalities were frequently cited as the context within which young women made the decision to engage in transactional sex, fashionable clothing and alcohol were mentioned more often than other items across all FGDs. The prominence of fashionable clothing and alcohol in young women’s lives was attributed to local definitions of modernity:

*…The modern girl here, she likes clothes***(FGD 1, young women aged 16–21)**

*… You see, in these times we are living in we like cars, we like clothes, we like alcohol, too much!***(FGD 3, young women aged 16–24)**

The meanings that young women attached to fashionable images were communicated during the exercise that we employed in the two FGDs where we invited participants to offer opinions about a printed image of a young, Black woman dressed in fashionable clothing. For the participants in FGD 3, the image represented a woman who is sophisticated, employed, wealthy or in a relationship with a sugar daddy who spends money on her. Participants in FGD 4 thought that the young woman in the image represented success, happiness, modernity, a love for clothes, financial independence, control, and strength.

For the participants then, by physical appearance alone, a young woman could convey important messages about herself, such as her affiliation to fashionable society, modernity, her economic background, and involvement in an economically rewarding sexual relationship.

In a context where fashionable images were laden with such important meanings, to be found lacking fashionable clothing was perceived as tantamount to social suicide, and was said to lead to social exclusion, feelings of shame and loneliness:

*…on the weekend there must be a kit (fashionable clothing that is determined by a peer group for a particular day/event) that you have to have. If you don’t have that kit… you hide yourself like at home because your friends are wearing new clothes and things like that.***(FGD 2, young women aged 16–18)**

Thus, young women displayed heightened awareness of others who were better dressed and well-off among them. They were said to use transactional sex and the money/material benefits generated from the practice to negotiate socio-economic inequalities in ways that proved that they were equal to, or the same as the next best dressed, socially mobile and economically well-off young woman in the community:

*And like, in our days, there are children who are coming from well-off homes, you see, they have all these clothes, so these ones feel small, so in an effort to look like the others they resort to this behaviour, to get money* (**FGD 5, men in their early 20’s and 30’s)**

*She has to dress up… she has to be similar to her friend. And it shows. Let’s say her [well dressed] friend is dependent on her parents, but she does not have parents at all, or she has parents but they are uneducated and stuff…*(**FGD 3, young women aged 16–24)**

According to some of the women, men willingly spent on their female partners’ dress and hair because they believed that a woman’s outward appearance had direct implications for her male sexual partner’s image and reputation in the community:

*[He] can buy me something to wear when the two of us are going out …he won’t want me to be seen looking snaaks (ugly) when I’m going with him, you see…***(FGD 2, young women aged 16–18)**

From the above, we see that the focus on fashionable images in this community was not only driven by the young women’s preoccupation with modernity, but by widely shared perceptions that linked outward appearance to reputation.

### Sugar daddies: popular, profitable, but scorned gateways to modernity and fashionable images

Young women reported a willingness to do whatever was required of them to access fashionable items of clothing, alcohol and money. While one young woman suggested that some might resort to “…taking an old lady’s social welfare grant”, others reported that transactional sex with older, wealthier men (locally referred to as “sugar daddies”) was a popular strategy for accessing cash to buy desirable goods and a lifestyle that was otherwise unavailable to many young women in the study community:

*…she has a sugar daddy, she asks her sugar daddy for money, she uses it to provide for herself, you see* ( **FGD 4, young women aged 16–24)**

*…he will buy you airtime, buy you everything…everything, [he will ask] "what are you in need of?"***(FGD 3, young women aged 16–24)**

*Because I am also driving now, because of my sugar daddy…I am driving now and I even drive to Cape Town, anywhere, because of my sugar daddy…***(FGD 4, young women aged 16–24)**

The young woman featured in the quote above was said to be the envy of all her friends because accessing driving lessons was deemed the height of modernity and quite uncommon for most young women in the study community. Thus sexual relationships with older and wealthier men were seen to hold multiple possibilities for young women to not only escape poverty, but also live out what was clearly construed as a modern and successful femininity.

Young women reported that the failure to obtain fashionable items from parents and friends led to only one other means of meeting a young, woman’s needs – sugar daddies:

Moderator: … so what happens if you get a parent who doesn’t have the money to pay or doesn’t want to?

Participant: Yes, they borrow the clothes [All answer]

Moderator: They borrow from whom?

Participant: From their friends

Moderator: Okay, so you borrow from your friends

Participant: Hmm

Moderator: What if your friends don’t lend you their clothes then, what are you going to do?

Participant: Sugar daddy, Sugar daddy [All answer, Laughter]

Moderator: So basically he is always ready to get these things

Participant: There is no other way

(FGD 2, young women aged 16–18)

In a culture where young women generally date men who are older than they are [47] and where material rewards are accepted as necessary markers of important sexual relationships [1,2,14,15,48], understanding the precise characteristics that distinguish a sugar daddy from an ordinary older, generous male sexual partner is not without complexity. However, it was clear from the conversations that we had with the young women that not every older, wealthier male sexual partner was considered a sugar daddy. In fact young women and men perceived a clear distinction between relationships with a sugar daddy and those with other types of sexual partners, including those who are older but not necessarily acquired for transactional purposes:

*It’s a sugar daddy when you eat his money…when you are not romantically involved, just eating his money…you are there to eat his money that’s all.****(*FGD 3, young women aged 16–24)**

Specifically, sugar daddies were considered to be older men that young women acquired solely for financial or material benefits. It seemed that the “sugar daddy” label was considered inappropriate in relationships with older men that were also characterized by romantic notions of love, even if the relationship featured monetary exchanges:

*Obviously, he will still give you money, that is fine but what I am saying is that we should not stereotype this sugar daddy thing; we should not make it [only] about age… Do not let it be, let it not be said that every young woman who is dating an older man is dating a sugar daddy***(FGD 4, young women aged 16–24)**

Men were especially concerned to point out that some age asymmetric relationships were not motivated by monetary exchange, but genuine sexual attraction:

… It’s like there are always two sides to an issue, you see, sometimes you find that she does not want money from you, she is just into you.

(FGD 5, men in their early 20’s and 30’s)

Despite the popularity of sugar daddies, young women were not altogether uncritical of this type of relationship. Some participants’ reports delineated the circumstances within which young women made the decision to date older men into categories of “right” and “wrong”. The circumstances that were considered “right” included instances where the young woman’s family was poor and thus unable to afford her the apparels that were associated with a life of modernity. “Wrong” circumstances included those where there was an absence of poverty and/or where the young woman was perceived to have alternative economic strategies:

*I can just study, I can fund my studies because my parents use their government social grants to the last penny for my education…so if you are dependent on a sugar daddy; that is wrong.***(FGD 3, young women aged 16–24)**

Further, in some young women’s discussions, sugar daddy relationships were associated with a number of negative factors. Such factors included ideas that having a sugar daddy who meets a young woman’s every need inspired complacency, compromised the chance of finding true love with same age partners, undermined dignity, self-esteem and self-efficacy, and exposed one to HIV:

*It’s wrong…because when you have a sugar daddy who is able to do everything for you, things that you are not able to do yourself or your parents are not able to do, nhe, you get that thing…you become complacent as a person***(FGD 3, young women aged 16–24)**

*She loses her self-esteem, her dignity…like among same age men, you lose your dignity because you went to an old person… secondly, once he gets sick (referring to HIV), you are done for***(FGD 4, young women aged 16–24)**

Participants in the men’s FGD boasted about their benefactor role as providers of plenty in the context of little in their sexual relationships with poor, younger women:

*You are an old guy, she has nothing, you are paying for her hair, you are buying her Truworths (popular clothing store), you buy her lunch and everything, you give her money, so that she has money for school*…**(FGD 5, young men in their early 20’s and 30’s)**

These men perceived their age and economic dominance in these transactional relationships as accruing for them unlimited power to control their female sexual partners and the terms of their sexual encounters:

*Because you are putting a carrot in front of her, she has to listen to you…whatever you want her to do, she will do***(FGD 5, men in their early 20’s and 30’s)**

*You have more power (laughs)…he is the one who is going to have an upper hand because the guy is older and he has more money***(FGD 5, men in their early 20’s and 30’s)**

Some of the women confirmed the gendered power imbalances that transactional sex with older wealthier men brought, but seemed to accept this as what was due to a woman who pursued men for money:

*You ate his money so he is controlling you***(FGD 4, young women aged 16–24)**

Within this context of a popular yet gender-disempowering and somewhat criticized behaviour, dating an older man purely for love was considered a luxury not available to poor young women who lived in environments of social and economic inequality:

*There is this idea that age is not important, that if you love a person, you love him, that’s all there is to it. But it happens that you are struggling at home where you are coming from and then your friends dress well, and if they want things from their parents, they get them, and yet you do not get those things, then you are forced to find a sugar daddy***(FGD 4, young women aged 16–24)**

Dating an older man merely for love was considered an indulgence available only to women from financially stable homes:

*Sometimes you…how do I put it…sometimes it is alright to date someone who is that old… because you love him, you do not care about his assets, because at home you are financially stable, you see.* (**FGD 4, young women aged 16–24)**

Thus, young women framed the decision to enter a relationship with a sugar daddy as one that was somewhat forced upon them by circumstances of economic deprivation in their homes, within a broader context of local economic inequalities and great pressure to uphold images of a fashionable and modern lifestyle.

### Weighing up priorities: material benefits versus sexual health

Notably, the young women’s conscious awareness of the negative aspects of relationships with sugar daddies was strongly contradicted by reports of their high prioritization of money and desirable goods over all else, including sexual health:

Participant: *As long as he satisfies your needs for money and alcohol you don’t really care. That is how it is around here…That’s what the slogan says, “Money Talks, Bullshit Walks”*

Moderator: *Okay, so what is the bullshit here that’s walking?*

Participant: *Bullshit is the HIV.*

Moderator: *Okay.*

Participant: *It’s what the people say.*

Moderator: *Okay including what other people say?*

Participant: *Hmm.*

Participant: *Yes.*

Moderator: *Alright, so those things don’t matter?*

Participant: *They don’t matter.***(FGD 1, young women aged 16–21)**

The above dialogue ensued when we were discussing the implications of transactional sex for the young women’s sexual health, reputation, and image. The participants made light of the negative sexual health outcomes of risky sexual encounters. When young women encountered opportunities for transactional sex, other matters including HIV, were “set aside” to make way for high monetary returns from their sexual encounters:

*Like, when a woman meets a guy here she does not think straight, especially not about HIV and other STDs, its only money, not diseases, she puts them aside and attends to the guy***(FGD 1, young women aged 16–21)**

In this context, extremely risky sexual encounters including descriptions of sexual encounters that seemed to suggest unprotected group sex, were dismissed as unimportant:

Participant*: Sometimes my sugar daddy will want me and then say bring your friends along so we can have fun, do you understand? He finishes (has sex with) all of us, he does not want to use condoms with me, he is not going to want to use them with my friends*

Moderator: *And how do young women feel about this?*

Participant: *No, you just eat his money, you go, you don’t care about that… you are there to eat his money that’s all.***(FGD 3, young women aged 16–24)**

The low regard for HIV was further pronounced by reports that the virus is rarely mentioned in conversations among young women and their sexual partners. In instances where it was mentioned in the FGDs, young women rarely referred to it by name, preferring to call it “the sickness”, and various humorous names that attempted to trivialize it.

### The convergence of new pressures, popular youth culture, and global technologies that enable a thriving environment for transactional sex

The popularity of transactional sex in the study community was said to be further fuelled by a new commodity that had become an important accessory for modern young women to possess: younger male sexual partners, locally referred to as “is’ today” or “is’ now now” (in reference to their youthfulness and associations with modern times):

*…they like to date younger boys…it’s fashionable… a young boyfriend dresses smartly, so it is something for status …they are called “is’ Today, “is’ now now” (in reference to his youthfulness, especially in comparison to sugar daddies)***(FGD 1, young women aged 16–21)**

Dating younger men was thus described as another important symbol of modernity among young women in this community. ‘‘Is ‘today’s” were defined as younger male sexual partners, often in their early to late adolescent years:

*I can be 19 but have a young boyfriend aged 13, 14…***(FGD 1, young women aged 16–21)**

Same age partners were also classified as “is ‘today’”. The greater likelihood that a young woman would have sexual relationships with older men was said to explain the description of same age male sexual partners as younger partners:

*He is your younger boyfriend even if you are the same age, becauseyou first got involved with older people***(FGD 1, young women aged 16–21)**

These sexual relationships were said to run concurrently with sugar daddy relationships. They provided young women with the economic ability to offer financial support to their younger male sexual partners, transforming their traditional position from beneficiary to benefactor in sexual exchange relationships:

*…you get money from your sugar daddy and then you share it with your young boyfriend (***FGD 1, young women aged 16–21)**

*…some will have an older sexual partner and a younger boyfriend as well***(FGD 3, young women aged 16–24)**

In this way, sugar daddy relationships were shown to have a place of importance in the young women’s lives. Young women’s relationships with older men yielded not only profitable rewards to meet subsistence and consumption needs, but also created opportunities for them and their younger partners to push social boundaries about what constitutes appropriate relationships.

Notably, the “is ‘today’s” were said to maintain these relationships for reasons similar to those that motivated young women to stay in transactional sexual relationships with older men, namely to access money, alcohol, and clothing:

*So he does not go anywhere, because he knows she is going to buy him clothes, his sugar mama is going to buy him everything he wants***(FGD 3, young women aged 16–24)**

Thus young women were said to buy and maintain the affections of their younger male sexual partners by ensuring that they were fashionably clothed, fed, and provided for during outings to shebeens.

Peer norms that inspired group behaviour among young women in the study community were reported to be another major driver of transactional sex and associated activities. Young women’s descriptions of peer norms included reports of weekend rituals, which involved young women informing their “schemes” (friendship groups) about the fashionable dress code of the day, meeting up, visiting the local shebeen, and once there “selling” each other to wealthy men for alcohol:

Moderator: *The scheme issue, I want to understand it. What is a scheme?*

Participant: *It is a group of friends… It works when you’ve noticed a certain guy who has money, then you call your friends …It is important because when you are going somewhere there must be a plan A as well as a plan B…Maybe today you are the one who’s being sold so you must seduce men… they give each other turns if this week it was Lindiwe, the following week its somebody else’s turn…***(FGD 1, young women aged 16–21)**

Young women did not censure the idea of women selling each other; they perceived it as just another necessary strategy to maximize their chances of guaranteeing a good flow of alcohol during outings to the shebeen. However, a good looking member of FGD 1 complained that these “selling” activities were sometimes exploitative, especially for the prettiest young women in these groups, who were sometimes “sold” more regularly than others.

Peer norms and dictates were said to extend beyond young women’s groups to include those that operated to define desirable masculinities and create peer pressure for men:

*You must drink, if you do not drink here in the township you are looked at as someone who is strange, you see my brother?* (**FGD 5, men in their early 20’s and 30’s**)

*If you are a man and you don’t have a car that means you are nothing here…* (**FGD 1, young women aged 16–21**)

…*in everything there is pressure, so even on your parents, you pressurize them, you say “I want this takkie (sneakers), I want this, I want that” you see?***(FGD 5, men in their early 20’s and 30’s)**

Importantly, not all the young women that we spoke to expressed positive regard for group norms; a few young women considered schemes to be unimportant and shared reports of jealousy and disloyalty in these groups. However, the general impression was that these peer norms were powerful in monitoring, appraising, and punishing peer behaviour that deviated from the local popular youth culture of maintaining fashionable images, drinking copious amounts of alcohol, and propositioning casual sexual partners for transactional sex purposes:

*If you cannot sleep around (i.e. engage in casual sex) you get kicked out of the scheme…* (**FGD 1, young women aged 16–21)**

*They pitsha (a local term used to describe visiting shebeens to meet new sexual partners for transactional sex purposes) so when you stay at home they look down on you and things like that… like they say bad things about you* (**FGD 2, young women aged 16–18)**

Further, these peer norms were said to be reinforced by a free, local web-based social networking forum, which young people in the community used to name and shame each other:

*We gossip about each other, there is even a web… the web where people are picked on, where it is said “so and so was such and such” how are you then supposed to feel?* (**FGD 2, young women aged 16–18)**

*They make fun of her and write about her on the web (***FGD 1, young women aged 16–21)**

*Last year I was discussed a lot on the web, a lot…of course I did not like it***(FGD 3, young women aged 16–24)**

Incidents that were said to be likely to surface on the social networking site included those about wearing unfashionable clothes or clothes that they had been seen wearing the previous weekend:

*If you wear the same clothes this week that you were seen wearing last weekend and the weekend before that, someone will take to the web and you know by the detailed descriptions of what you were wearing, that they are talking about you…everybody laughs and mocks your clothing…***(FGD 2, young women aged 16–18)**

Thus, the fear of socially punitive consequences for those who did not conform or were found lacking in terms of fashion and drinking, placed real pressure on young women to invest in fashionable clothing and to engage in the activities linked to popular culture.

When asked to mention factors that influenced their sexual risk behaviours, young women cited television images, modern technology, and local and international young, Black, successful female celebrities. These celebrities were said to put young women “under pressure” to pursue images of wealth, style and success through sexual relationships with older, wealthier men:

*Yho! I´d say it’s the television! It’s the television, its technology…***(FGD 3, young women aged 16–24)**

*…we are put under pressure by Khanyi Mbau (a local celebrity famous for a fashionable lifestyle that is entirely funded by her sexual relationships with older, wealthier men) it’s them that corrupt us***(FGD 3, young women aged 16–24)**

Interviews with community members who were aged 16 years and older in the late 1980’s suggested that the availability and variety of commodities had increased compared to 15 years ago, and that this was related to the commonness of transactional sex among young women:

*…It’s because everything is available or you know technology as well because 15 years ago there were no such things as MXIT (a very popular and cheap social networking facility available on mobile phones) 15 years ago there was not much* [access to] *TV***(Community member, local nurse)**

*There are too many commodities…they overwhelm our children***(Community member, housewife)**

In addition to these ideas of increased availability of commodities, there were reports that 15 years ago, there were fewer goods. Interestingly, one community member linked the perceived substantial increase in commodities and the proliferation of televised images to the post-apartheid era, suggesting that ordinary South Africans link this critical period in the country’s history to the onset of the more pronounced presence of globalization:

*The first thing is TV…these things started in the late 1980’s but really excelled after 1994***(Community member, taxi driver)**

According to these community members, very few young women were able to afford fashionable images and those who could, either came from well-off families or were likely to have accessed their clothing through a phenomenon known as *ukuminca,* which refers to holding (and hiding) items stolen from department stores between one’s thighs:

*Back then you really had very few clothing shops…they were expensive, yho! … if you saw a girl who is always wearing fashion*[able clothing] *you just knew this one she practices ukuminca* (laughing) *or maybe her sister or her aunt…unless they have everything at home***(Community member, housewife)**

According to these community members, there were visible changes in the consumption patterns of young people in this community. These changes were informed by significant shifts in the availability and pricing of goods and the population’s exposure to global images and technologies, so that what was previously uncommon became in abundant supply.

## Discussion

The findings from this study suggest that transactional sex was perceived as a lucrative economic strategy among young women in the study community. It was a practice that represented opportunities to access plenty in the context of little. Through transactional sex the young women could meet their subsistence and consumption needs, attain symbols of modernity and successful womanhood, purchase admission into social groups, adopt new and fashionable gender roles as partners to and providers for younger sexual partners, conform to the dictates of popular youth culture, and avoid social exclusion. Despite these perceived benefits, the risk behaviour was also shown to create opportunities for the domination of young women by their older and wealthier sexual partners similar to that found in studies from other parts of South Africa [8,15]. In the young women’s accounts of their transactional sexual relationships with sugar daddies, it was also clear that their sexual encounters were sometimes unprotected, and extremely risky, thus heightening their vulnerability to HIV. Latent analysis of the findings suggests that in the study community, contextual factors that created the high demand for transactional sex included the pursuit of fashionable images, popular youth culture, globalization processes such as the increased availability of commodities and technologies in South Africa, limited economic opportunities, inequalities and poverty.

Owing to the multiple ways in which transactional sex was used among the young women, the items for which they exchanged sex were varied: from food and electricity, to alcohol, fashionable clothing, and cash. As has been found in other studies, [2,17,38] fashionable commodities were crucial to young women’s social identities in the study community. This finding may be indicative of the need to re-examine notions of what women in poor communities need to survive. Food may be but one in a range of essential items that they must have in order to achieve perceived success in their society.

Redefinitions of survival have implications for how data on the items that are exchanged in transactional sex are used to theorize about what level of poverty motivates women to engage in the practice, and whether the items reflect survival or consumption sex. To deduce consumption versus survival motivations for transactional sex, the prevailing practice has been to classify the items of exchange (such as clothing, food, school fees) that participants name in their accounts about the practice into needs versus wants; basic versus consumption items [2,18,49] despite giving no indication that participants personally reported any hierarchical categorization of these items. And yet, constructs of luxury and necessity are specific to individuals, and as such vary from person to person depending on their social position and the variety of goods to which they are exposed [50].

When we engage in this hierarchical categorization of items for which poor women exchange sex, we run the risk of misclassifying what they consider important needs as frivolous luxuries. Consequently, we may design inappropriate interventions since we would have misconstrued the true underlying motivations of their sexual risk behaviours. From these findings we see how the pursuit of fashionable clothing was not a mere exercise of consumption for consumption’s sake, but one that was necessary for surviving social exclusion, loneliness, and scorn. Further, it is well-established that along with investing efforts in strategies that ensure the survival of basic hunger, “even the poorest people engage in conspicuous consumption” [51]. Thus, a more meaningful consideration of the motivations for transactional sexual relationships is one that considers that they are varied, arising from and meeting diverse needs among women for whom “the only economic capital some of them possess is their sexual organ” [1].

Young women’s preoccupation with fashionable clothing was not unexpected since adolescence and young adulthood are developmental stages where most young people are highly sensitive to their outward appearance and the perceptions of their peers about it [35]. Further, the aesthetic, social, and economic meanings that the young women in this study attributed to fashionable images suggest ways in which the women in our study used their own outward appearance/s to convey positive perceptions and attributes about themselves. This is not unique, as clothing is generally used as a communication tool, which “allows a message to be created and [selectively] understood” [52]. Further, young women’s perceptions that fashionable images conveyed important statements about one’s socio-economic background and personal attributes are indicative of consumerism, a phenomenon that is “above all else a coded system of signs [messages through images], through which people communicate with each other” [53]. This being the case, it is not surprising that clothing held a place of such importance for young women in this community, because consumerism transforms goods into necessities that become useful in how we construct our identities [54]. The easy accessibility of large and varied quantities of fashionable goods that were previously unavailable or available only in limited quantities in township locations during the apartheid era [44] may have transformed young women’s roles as consumers. Previously, communities such as the study setting were isolated from mainstream economic development and had limited access to information, commodities, and knowledge about the lifestyles of those beyond their immediate surroundings. Through the lifting of restrictions on Black people’s movements, improved road and transport infrastructure, the influx of goods from Asian markets, and increased and unlimited access to local and global print and visual media in the post-apartheid era, previously “landlocked” townships suddenly opened up socially and geographically. These macro-level changes may have set the scene for conspicuous consumption of the highest order among a population made vulnerable by their limited economic power to benefit positively from globalization. Further, the young women’s interaction with globalization suggests that among the poor the phenomenon has simultaneously created opportunities for inclusion and exclusion from the global arena. Inclusion opportunities have materialized in that the images and goods that were once remote have been brought near, but due to the lack of economic capacity to access these commodities, many poor populations are “left behind” [55].

To explain their obsession with modern images of success and their use of transactional sex to access them, young women blamed global and local media images as shown on television. These reports confirm widespread notions of the media as consumerism’s most loyal and rewarding handmaiden, used by “brokers of consumption to construct the everyday reality of consumers” [54]. Further, young women’s reference to a celebrity’s influence on their obsession with fashionable images indicates a number of important issues. First, these young women were not ignorant consumers of media images of success and wealth; they were able to reflect on the way in which they were affected by what they viewed on television. This suggests that there are opportunities to use the media to create alternative and helpful HIV risk-sensitive images and narratives for young women to absorb. Second, it serves to confirm the long established theory of emulation, where consumers of a lower class seek to establish similarity with those above them in the social hierarchy by vying for images and habits that are found among the wealthy [56]. While in the 19^th^ century, the lower class’s exposure to consumers within higher social hierarchies would have been limited only to those visible in their immediate surroundings, in modern day South Africa the process of emulation is able to occur with greater intensity with the aid of the media and modern technology inherent in cultural globalization.

The existence and widespread use of the community’s web-based social networking tool was further testimony to the extent to which these young women and the rest of the community were “global citizens in local states” [57]. People around the world are increasingly exposed to social networking technologies, which simultaneously expand and shrink their worlds, so that they are at once extending their social networks and yet reducing social spaces in which they can “hide” and live private lives. In this community the use of social policing tools that made transactional sex necessary for young women to survive social exclusion, gossip, and scorn created additional pressure to conform to peer norms of consumption and the upholding of fashionable images.

The findings about young women’s awareness and pressure to emulate those who are better off are instructive about the use of transactional sex in societies that are not only poor, but also defined by local economic inequalities. Even though the study community continues to exist as a poverty pocket within a wealthy region, intra-racial economic inequalities that exist within many Black African communities in modern day South Africa [58] are mirrored here. As such, beyond affording young women with capital to prove their agency, increase their desirability and popularity among peers, sexual exchange relationships with sugar daddies were used as one way of bridging the social and economic inequalities that existed in the study community. However, sexual relationships with older men place young women at high risk of HIV infection [6,9,59-61]. Evidence on age mixing suggests that a young woman’s risk of becoming infected with HIV is significantly increased by an age difference between herself and her older partner of as little as five years [9]. In this study, the sexual control wielded by older men further increased the young women’s vulnerability to HIV.

Young women’s criticism of sugar daddy relationships suggests that, had they possessed other means of accessing the items of exchange that they acquired through transactional sex, the practice would not have enjoyed the high level of popularity that it did.

Sexual exchange relationships between young men (adolescent males) and older women who are employed or in profitable sexual exchange relationships with older men, is uncommon in Sub-Saharan Africa [59,62]. The phenomenon, which was evident in the study setting, suggests a number of things about the use of transactional sex to reconfigure sexual norms. First, it might be a reflection of a shared perception that transactional sex is a highly lucrative strategy for both young women and men who have opportunities to use their sexuality to link up to older, wealthier patrons. Second, it suggests a possible shift in African constructions of the dating and mating game, which generally censure intimate relationships where the male partner is younger than his female counterpart. Third, this shift may be indicative of opportunities for change in other gender norms that are discriminatory towards women and those that do not serve women’s reproductive health interests. Fourth, the provider role that is played by young women who are in sexual relationships with younger male partners can be interpreted to suggest two major functions that stand in tension with one another. On the one hand it serves to create possibilities for avenues of power (sexual, economical and decision making) otherwise unavailable to young women in sexual relationships. On the other hand their provider role may be reinforcing the common status of women as primary caretakers, nurturers, problem bearers, and breadwinners in many Black African families in South Africa. Finally, young women’s concurrent sexual relationships with older and younger men produce “concurrency superhighways” [63] that create fertile opportunities for HIV transmission across age cohorts, from older men to young women to their even younger male sexual partners.

Another uncommon finding of the study concerns the young women’s reports that they sometimes “sold” each other to guarantee access to alcohol during outings to shebeens. It suggests both the extent to which young women might be prepared to go to satiate their appetites for symbols of modernity, as well as the capacity for young people’s friendships to become contexts of risk and possible coercion [64]. On the other hand, peer groups that foster pro-social and positive behaviour have been associated with young people’s reduced vulnerability to risky behaviours [65].

Throughout data collection young women’s conversations about HIV were marked by a casual attitude towards the virus and a trivialization of sickness and disease. The young women’s casual, dismissive attitudes towards HIV invite reconsideration of the vulnerability paradigm that is often used to explain women’s higher susceptibility to HIV infection. This paradigm assumes that women (and not men) want to prevent HIV and that the main barrier to safe sex practices is their male partner’s negative attitudes towards condom use [66]. Owing to this paradigm, HIV prevention theories and programs concentrate on the importance of empowering women to insist on condom use, rather than understanding whether they even believe HIV is important enough to protect against, and what their feelings about using condoms are. More useful approaches are ones that see the establishment of our understanding of women’s individual perceptions of HIV risk and their personal regard for condom use as an important point of departure in HIV prevention.

Further, the young women’s casual regard for HIV suggests that the virus was not considered the most urgent threat to circumvent [22], as judgment and social exclusion by peers were perceived to pose a greater hazard. This may be explained by the fact that young, socially and economically vulnerable women have less incentive to forego the rewards of risk behaviour that are linked to financial and material gain [67]. In the context of poverty and inequality, the poor are also known to engage in riskier behaviour as social values are more easily eroded by stressful circumstances of deprivation [50]. It may also be that when one is conditioned to a life surrounded by multiple risks (e.g. risks of violence, sudden loss of income or the death of key relatives), risk becomes normalized and thus not urgent to avoid.

### Limitations

Our study has a number of limitations. First, we did not enquire about participants´ individual socio-economic status, and so this paper has had to rely only on the study setting’s demographic profile to deduce the participants’ economic status, which overall was low. Second, the findings of the paper are largely based on data from FGDs, which are known to sometimes produce only the single stories of the group’s most dominant participants, rather than multiple and contesting realities. We attempted to overcome this by conducting several FGDs with different types of social and age groups. Third, our sample is in no way representative of the whole population of young women in South Africa or in the study community. However, the participants´ descriptions do provide important insights about those women who engage in transactional sex in the study setting and others similar to it. Fourth, we limited the IIs to six participants because the decision to include the perceptions of community members in the enquiry was only made towards the end of the data collection period. By then, we were approaching the festive season (November –January) a time that is difficult for research in Black communities because many families migrate to the rural Eastern Cape Province for the holidays.

## Conclusion

Our findings suggest that young women in contexts of generalized poverty in modern South African townships engage in transactional sex to meet needs that are necessarily varied. Transactional sex is perceived to be a lucrative economic strategy that allows young women to not only ward off hunger, but to access the fashionable images that are necessary for social survival. Put simply, cash incentives from transactional sex allow them to access what young people in many parts of the world also prioritize. Innovative strategies that address the structural realities that make it difficult for young women in poor communities to choose a different gateway to images of modernity and avoid social exclusion from their peer groups are needed.

The findings also show that young women are able to use transactional sex to carve out new gender identities by using proceeds from the practice to take on provider roles in sexual relationships with younger men, suggesting new possibilities for gender equality in poor communities. Future research is needed to exploit these possibilities and assist women in the creation of protective and empowering gender roles. Finally, more research is needed to explore how best to foster a sense of urgency and priority around HIV among young women and men who live in contexts of poverty, inequality and exposure to multiple risks.

## Competing interests

The authors declare that they have no competing interests.

## Authors’ contributions

YZ, LT, AT and AME conceived and designed the study. YZ, LT, AT and AME developed the study protocol. YZ developed the data collection instruments. YZ collected the data. YZ and LT analysed the data. YZ, LT, AT and AME drafted the manuscript. All authors read and approved the final manuscript.
